# The Climate of Aspley Guise
*Observations on the Topography and Climate of Aspley Guise, Bedfordshire, in Reference to their Influence on Health and Disease. By James Williams, M.D. London: Richards. 1856.


**Published:** 1857-01

**Authors:** 


### THE CLIMATE OF ASPLEY GUISE.*

DR. WILLIAMS has here given a very pleasantly written and interesting book. He makes out a good case for Aspley Guise as a residence for invalids, and shows that England has in herself the advantages which sick tourists seek abroad. The mean annual temperature of Aspley Guise is 47½°, the range of temperature 8°; the annual fall of rain is 18½ inches. The stratum is of sand; the water is good: and the mortality is from fourteen to fifteen per thousand. Dr. Williams's book may be perused with advantage by those who wish to select a residence, permanent or temporary, for the debilitated and consumptive.

We have several other books on our table, which for the present we must pass over with brief notice. Mr. Gamgee's *Researches*† are excellent, and add new credit to their able author. Dr. Routh's extended paper on *Fæcal Fermentation*‡ deserves the same praise. Dr. Leared's paper on *Phthisis*§ is a valuable statistical document. Dr. Lombard's *Climats de Montagne*|| is an instructive work, shewing the physiological effects of various mountain climates. Dr. Hyde Salter's *Introductory Address*¶ contains some excellent advice to students, given in plain language. Dr. Barnes's *Report of the Sanitary State of Shoreditch*, and Dr. Ballard's on *St. Mary, Islington*, will claim further attention. Dr. Stark's *Meteorology of Scotland*, for the June quarter, is compiled with much care and industry.

---

* Observations on the Topography and Climate of Aspley Guise, Bedfordshire, in Reference to their Influence on Health and Disease. By JAMES WILLIAMS, M.D. London: Richards. 1856.

† Researches in Pathological Anatomy and Clinical Surgery. By JOSEPH SAMPSON GAMGEE. London: Baillière. 1856.

‡ Fæcal Fermentation as a Cause of Disease. By C. H. F. ROUTH, M.D. London: Richards. 1856.

§ An Analysis of a Hundred and Thirty-Six Cases of Phthisis. By ARTHUR LEARED, M.D. London: 1856.

|| Des Climats de Montagne, considérés au point de vue Médicale. Par le Docteur H. C. LOMBARD. Genève: 1856.

¶ Introductory Address delivered at Charing Cross Hospital, October 1, 1855, on occasion of the opening of the Twenty-Fourth Session of its Medical School. By HYDE SALTER, M.D. London: 1856.

